# Indigenizing and Ruralizing NCI Screen to Save Program: Resources, Optimizing Outreach, Teaching, Science (ROOTS)

**DOI:** 10.1007/s13187-023-02376-8

**Published:** 2023-10-12

**Authors:** William Maybee, Whitney Ann Henry, Dakota Lazore-Swan, Corrinne Abrams, Kris Attwood, Paula Vincent, Kayla Catalfamo, Rodney C. Haring

**Affiliations:** 1 Department of Indigenous Cancer Health, Roswell Park Comprehensive Cancer Center, Buffalo, NY USA; 2 Department of Biostatistics & Bioinformatics, Roswell Park Comprehensive Cancer Center, Buffalo, NY USA; 3 Department of Cancer Prevention and Control, Roswell Park Comprehensive Cancer Center, Buffalo, NY USA

**Keywords:** Colorectal, Cancer, Screening, Health intervention, Prevention, Education, Outreach, Indigenous, Native American, Rural, Patient navigation

## Abstract

Colorectal cancer (CRC) is a complex health disparity in many Indigenous and rural populations. While it affects anyone regardless of race, age, gender, or other common differences among people, Indigenous and rural populations are at a higher risk of dying from colorectal cancer. An NCI Screen to Save (S2S) program was culturally tailored to promote awareness and knowledge of colorectal cancer and screening in both Indigenous and rural communities across a sector in Northeastern USA. Indigenous and rural community outreach teams at an NCI-designated cancer center partnered with a community advisory board to provide an indigenized/ruralized version of the NCI Screen to Save program delivered to both Indigenous and rural/suburban communities. In total, *n* = 79 pre/post surveys were obtained from *n* = 82 participants, who had an average age of 49 years. Findings demonstrated that Indigenous/rural participants in both off-territory/non-reservation communities and a tribal community that received a culturally tailored version of NCI’s S2S program were able to identify both smoking and tobacco use along with lack of physical activity as risk factors for colorectal cancer. Post-intervention, participants reported being more likely to increase physical activity. Most importantly, participants said they would be more likely to be screened for colorectal cancer along with their family and friends based on their cancer screening experiences. Culturally tailored CRC messaging is an effective means for increasing screening intentions and decreasing cancer health disparities among both indigenous and rural populations. Future research should include the relationship of diet to obesity-related cancers, greater integration of Indigenous-rural patient navigation programs, creation of more information on genetic screening, and quality improvement to service translational science initiatives.

## Introduction

Colorectal cancer and cancer health disparities are concerns in rural and indigenous populations. For this paper, Native Americans, First Nations, Alaska Natives, Native Hawaiians, and American Indians are noted as Indigenous. Colorectal cancer (CRC) is one of the most prevalent cancers in the USA. In the Roswell Park catchment area, CRC is the fourth most diagnosed cancer in the region [1, 2]. While it affects all people, Indigenous people are at higher risk of dying from colorectal cancer [3–5]. 4.3% of American Indian/Alaska Native (AI/AN) people are likely to develop CRC. The disparity in these figures is pervasive due to underreporting by governing agencies driven by racial misclassification in electronic medical records (EMR) and large variance in CRC based on geographic location [3]. Alaskan Natives, for example, have the highest rates of CRC in the world because of their remoteness and limited access to cancer screening and treatment [6]. However, access to care in remote areas may only partly explain the disparity [7]. Lower screening rates and higher CRC rates are attributed to many factors, including masculinity barriers and medical mistrust [8]. Indigenous masculinity barriers are closely correlated with decreased CRC screening intentions [9]. Other screening barriers include language, discrimination, cultural insensitivity, fear, discomfort, and screening preparation [10, 11]. The lack of engagement in cancer prevention education may also be a contributing factor. Haring et al. analyzed cancer death rates and leading cancer causes of death for the Haudenosaunee Nations (ancestrally related tribes in NY State) and then compared these Indigenous populations with White persons by sex from 1990 to 2009. Findings indicated that CRC was the second leading cause of cancer death among the Haudenosaunee [12].

Healthcare systems often lack the infrastructure to adequately screen for preventable cancers in Indigenous, rural, and other minority populations. This may result in lower screening rates and subsequently later-stage cancer diagnosis. Thus, it has the potential to increase mortality rates [13]. A culturally adapted early screening program can help overcome screening barriers and increase treatment success and survivorship rates. In the past, screening guidelines were standardized for general populations. Recently, however, the increased participation of minority communities in colorectal cancer research and the lower average age of colorectal cancer diagnosis concluded that revisions to these guidelines were necessary. These changes include a reduction in screening age from 50 years of age to 45 years for all adults as of 2021 [14].

The Screen to Save (S2S) initiative was implemented in Indigenous and rural populations in collaboration with the National Outreach Network (NON). The NON is a national network of Community Health Educators (CHEs) aligned with the National Cancer Institute (NCI)–designated Cancer Centers (CCs) [15]. A culturally adapted S2S program was implemented and titled Resources, Optimizing Outreach, Teaching, Science (ROOTS) and was led by an Indigenous community health educator (CHE) with the guidance of Indigenous and rural community advisory board (CAB) members. We sought to explore whether a culturally adapted S2S CRC program altered CRC knowledge and screening intention in a cohort of Indigenous and non-Indigenous rurally-based individuals.

## Materials and Methods

S2S is an initiative with national implementation at the macro level by the S2S Collaborative with a common goal to increase awareness and knowledge of CRC and promote the uptake of US Preventive Services Task Force screening recommendations for all people 45 years of age and older. This project was conducted in the Northeast USA and encompassed engagement with rural and Indigenous urban, suburban, and rural populations with the guidance of an Indigenous community advisory board. The CAB was responsible for approving all cultural adaptations of S2S material to ensure appropriateness and accuracy. An institutional review board (IRB) approval was also obtained to expand the study beyond the RP catchment area. Subsequently, any research activities were also approved by the tribal governments of sovereign Native Nations. Deidentified survey data were collected and stored in an encrypted survey database. An indigenous cancer research team in collaboration with the cancer center’s biostatistical team provided the sole oversight of the data collected in the study.

The first phase of the project involved engaging current and establishing new Indigenous and non-Indigenous not-for-profit organizations to jointly create a “toolkit” that included (1) a one-page overview, (2) an Indigenous-specific and rural outreach plan template and strategies for engagement, (3) an Indigenized and ruralized educational/intervention presentation with key culturally and regionally attuned CRC messaging, (4) a pre- and post-educational/intervention activity survey with instructions for data collection, and (5) an Indigenized and ruralized NCI-approved general CRC messaging that was disseminated through an NCI-Designated Cancer Centers and Department of Indigenous Cancer Health via outreach. This included tribal newsletters, social media, and attending tribal health fairs using large displays (e.g., inflatable colon). The COVID-19 pandemic prevented most in-person research activities. Intervention staff and CHE in turn developed means to present the intervention virtually, including virtual conferencing with area leaders on and off reservation territories.

### Instrumentation

The project was delivered in a hybrid fashion (virtual and in-person when allowable) due to COVID-19 pandemic restrictions and social distancing guidelines. In-person education intervention was conducted using paper surveys and while projecting the presentation on a shared screen. The method of presentation delivery depended on the size of the classes, varying from one to twenty individuals. Virtual delivery was conducted using multimodal virtual platforms and face-to-face video communication (e.g., Zoom), Twilio (an SMS engagement platform), and REDCap (an encrypted survey distribution tool). Seventy-six percent of study participants chose virtual education; the remaining 24% chose in-person. Integrating the Twilio application within REDCap allows the study interventionist to send and collect survey data and engage study participants with future SMSs. Once participants connected with the CHE either in person or virtually, they first consented to the study prior to data collection. A 23-question demographic/family history and 19-question pre-educational/intention survey were then sent to their cellphone via SMS or email address depending on preference. Most participants completed surveys on their cell phones. In-person sessions, offered pre and post-pandemic, used the same structure and surveys. They chose to either fill out paper surveys, or have surveys sent to their cellphone. Once completed, the presentation was administered, followed by a post-education/intention survey that was again sent to the participant if conducted virtually. Pre/post-education surveys contained identical questions to assess changes in CRC knowledge and CRC screening intentions post-intervention. The average total time to complete the intervention ranged from 45 to 60 minutes.

The primary intervention materials included a culturally adapted presentation and CRC infographic administered in the same fashion regardless of virtual or in-person delivery. The key concepts within the presentation materials remained unchanged. In addition, based on Muller et al. [16] and Ocvirk et al. [17], following the intervention session, (1) culturally appropriate SMS was sent once per month for 3 total months encouraging CRC-eligible participants to consider screening.

The ROOTS program administered pre/post surveys to evaluate the effectiveness of the S2S health intervention program. After completion of the ROOTS program, participants connected with the NCI-designated cancer center’s Indigenous and Rural Patient Navigation program to continue conversations on screening, treatment, survivorship, and palliative care. The data included in this analysis focus on pre- and post-test results on basic cancer education and prevention messaging. In addition, five items assessed intentions to speak with a healthcare provider, get screened, talk to family and friends about CRC, eat healthier, and increase physical activity. The true/false items covered the knowledge of screening methods and guidelines and risk factors for CRC, including heritability. The surveys also contained information regarding participant demographics, CRC screening history, family history of CRC, and intentions to engage in healthful behaviors regarding CRC screening and prevention. Existing instruments found in previous peer-reviewed work formed the framework for survey questions [15].

### Participants

The community-based sample was recruited by the Indigenous research team CHE, a comprehensive cancer center’s Department of Indigenous Cancer Health team member, Indigenous/rural community advisory board, in collaboration with the cancer center’s creative services and marketing department. The research component involved a partnership with off-territory Indigenous and allied rural communities with a geographic emphasis on the cancer center’s catchment area counties (9 counties), rural and suburban landscapes, and partners adjacent to the reservation/territories. The initial participating Western New York (WNY) tribe went into a global research moratorium. However, another tribe in Northern NY approved in-person and virtual research activities in phase two of the study. Of the eighty-two participants, 40% identified as Indigenous, with the remaining participants identifying as more than one race.

## Statistical Methods

The analysis conducted represents a summary of preintervention activity observations. The survey responses summarized pre/post-intervention activity using frequencies and relative frequencies. The calculation of the mean and interquartile range (IQR) summarized continuous variables. Associations were compared using a generalized linear mixed method design (GLME). All analysis was conducted in RStudio v4.0.2 with a *p*-value of ≤ 0.05.

## Results

After receiving an Indigenized/ruralized intervention session, participants were significantly able to identify that smoking and tobacco use were risk factors for CRC (*p* = 0.0299). Furthermore, participants were able to identify that lack of physical activity was a risk factor (*p* = 0.0115) and reported that they were more likely to increase physical activity following the intervention (*p* = 0.01849). Participants were unaware that if they did not show symptoms, they may still have colorectal cancer (*p* = 0.01082). Significant changes in pre/post scores indicated that participants were more likely to be screened for colorectal cancer (*p* < 0.001) after the intervention and more likely to talk about colorectal cancer with family members and friends (*p* = 0.01123). However, there were no pre/post changes in knowledge, specifically that a poor diet or being overweight are risk factors for CRC. The results also did not show a significant change in knowledge related to how chances of colorectal cancer are higher if an immediate family member has it or has had it.

## Discussion

ROOTS is an outreach and intervention program focused on colorectal education and screening. An Indigenous and ruralized version of the S2S program collaborated with Indigenous and non-Indigenous people in and across rural, suburban, and off-territory not-for-profits along with grassroots organizations serving Indigenous and non-Indigenous populations. ROOTS provided a culturally tailored presentation, videos, screening information, and resources. A focus was on small media to recruit participants and engage study participants, such as short CRC vignettes, infographics, and fact sheets. Small media is proven to be a cost-effective evidence-based intervention for increasing CRC screening [18]. Our results indicate the importance of culturally tailored education materials and increased CRC screening intentions in a logistically feasible manner. A pre/post survey was conducted to determine areas of knowledge and behavior change. The most important intervention-to-action result was that a considerable number of Indigenous and rural community members said they were more likely to be screened and to talk with their family and friends about the experience post-intervention. Results showed that the ROOTS program helped participants identify smoking and tobacco use coupled with the lack of physical activity as risk factors for colorectal cancer. However, there are areas that could be fruitful endeavors in future investigations. This includes the need for more information on heredity cancers and the need for culturally adapted genetic screening protocols. The non-significant findings also shed light on the need for an increased conversation on co-occurring conditions such as obesity and cancer relationships and an emphasis on incorporating research into service programs toward co-occurring condition interventions. Also of note was the passage “I am likely to talk to my healthcare provider about colorectal cancer screening.” This did not meet statistical significance in testing, which relays the continued need for community-based patient navigation that is culturally relevant and locally significant.

We identified limitations to the study. The project had an IRB-approved protocol with community advisory board oversight. However, the initial tribe identified for the program went into a tribal-wide research moratorium. Members from the tribal community interested in the culturally tailored project were referred to general education, prevention, and public programming. The project in WNY was subsequently refocused toward rural and suburban rural areas: Indigenous and non-Indigenous with residence off-reservation or Territory. Tribal diversity lent itself to challenges in culturally tailoring educational material. Unique features such as different languages, geographic locations, and cultural norms made it difficult to capture the experiential features of each participating tribal member and rural community. Universal Indigenous and rural themes were woven into the content, but this may have compromised its potential relevance as a result.

The cohesiveness and diversity of educational delivery mechanisms were a strength of the study. Our Native and rural communities often reside in remote locations without access to existing or reliable Internet services or community gathering sites. Spurred by the COVID-19 pandemic, through a series of IRB amendments, we added multi-platform intervention delivery systems that included in-person, virtual (Zoom), or phone calls to meet the needs of individuals. We were able to maintain the continuity of the educational program to uphold scientific rigor and fidelity despite these challenges. Flexible programming is a vital component of successful health research in Indigenous and rural communities as the landscape may have more needs than urban settings. Another notable strength was the percentage of participants who identified as Indigenous or mixed-race Indigenous. Over 40% of study participants met that criterion. Historically, Indigenous participation in research and in particular clinical trials is low. We attribute our strong study numbers to well-developed partnerships with Indigenous communities and organizations that act as a social conduit to their respective communities. It should be noted that the development of this social capital requires many years of ongoing commitment. A significant amount of time allocated to the ROOTS intervention was spent cultivating trust with our partners and honoring the needs of their communities by providing non-research-related activities and services.

Future investigations should include extending the program to other tribal/rural dyads across the USA and increasing sample sizes with larger tribal/rural cohorts. Recruitment in future studies may also start intervention curricula at age 40 for research and recruitment purposes to educate and prepare community members for screenings at 45. Our study found that simple education programming may have a positive impact on CRC screening intentions. The work of Davis et al. further supports the notion of accessible health information as a key factor in improved health outcomes for underserved communities [19]. Service integration such as cancer-specific patient navigation is key to the success of early screening programs and the health of future generations. These are all part of the “medicine” and care for current and future generations.

## Data Availability

Tribal governance dictates our use of any data sets containing personal information from Indigenous study participants. Outside of the Indigenous research space, this practice is poorly understood. To summarize, our team must first obtain an agreement to conduct research on Tribal lands from Tribal government officials, or Chiefs. With the understanding that the Tribe has ultimate authority of any and all information collected. All other uses of that data is prohibited. Any use outside of the Tribal agreement is considered an infringement on Tribal Soverignty. As stewards of this data, we are obligated to protect it and restrict its use to extraneous entities.
